# From setback to comeback: navigating exam failure in medical training

**Published:** 2025-10-29

**Authors:** Nicola Quilter, Sara Lavarone, Kukuh Prasetyo, Juliet Mulenga

**Affiliations:** 1 Director of Examinations: Ophthalmology Foundation, UK.; 2 Person Centred Counsellor, Buntingford, UK.; 3 Ophthalmologist: JEC Eye Hospitals and Clinics, Purwokerto, Indonesia.; 4 Ophthalmologist: University Teaching Hospitals, Lusaka, Zambia.

**A failed ophthalmology exam is not proof that you are not good enough – it is feedback about what you need to do differently next time.**

**Figure:**
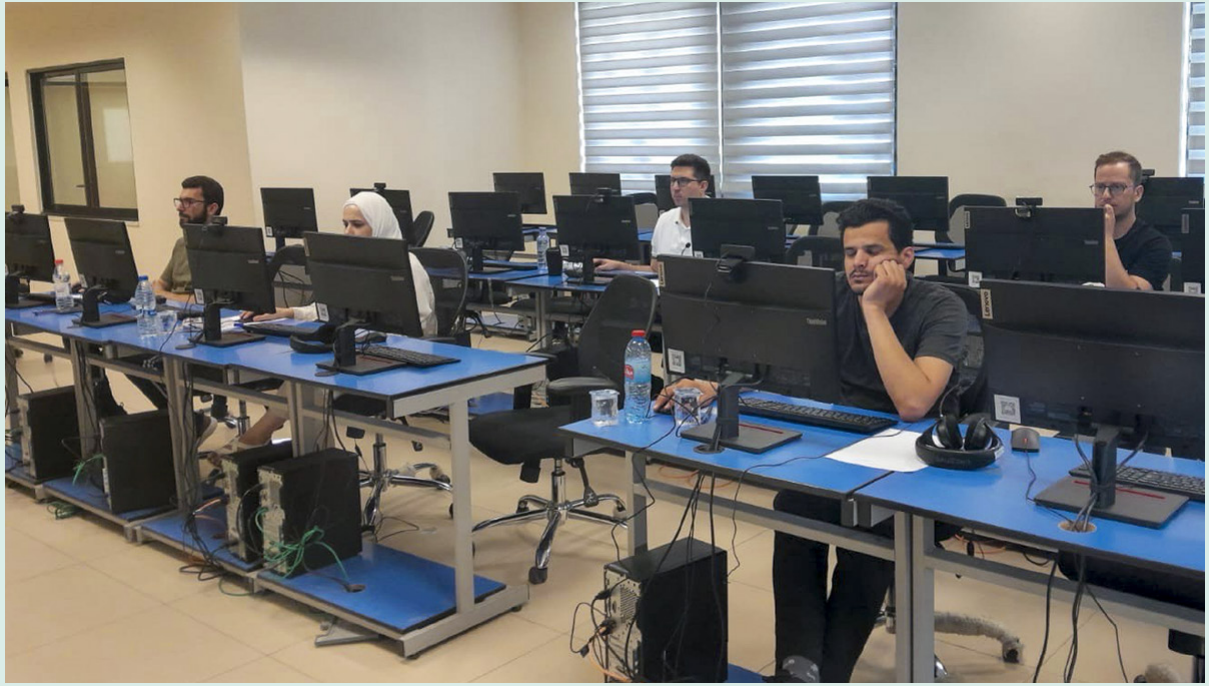
Candidates taking an online ophthalmology exam. JORDAN

© OPHTHALMOLOGY FOUNDATION CC BY-NC-SA 4.0

Failing an ophthalmology exam can feel like the end of the world: it is upsetting and frustrating, and some of us may even feel embarrassed, especially if we have worked hard and hoped for a different outcome. However, a failed exam does not define who you are or what you are capable of, and the result is not proof that you are not good enough – it is feedback about what you need to do differently next time.

Setbacks are inevitable in training, but they do not mark the end of your journey; any mistakes are learning opportunities. They also provide an opportunity to build resilience and confidence.

Here are our top tips if you have failed an exam:

- Avoid comparing yourself to others; focus on your own progress instead. Failing is part of learning.
- Be kind to yourself and allow time to process your feelings, including disappointment.
- Reflect on your experience, and what you can do differently when you retake the exam. Identify if nerves, insufficient preparation, or individual topics were specific challenges for you.
- Seek guidance and support from mentors or the examinations team of the organisation offering the exam. You are not alone!

Retaking the exam demonstrates determination and resilience. Before retaking an exam:

- Ensure you have had adequate rest and sleep, and take regular breaks while studying to enhance memory and focus. Incorporating short walks or mindfulness activities in your breaks may help to reduce anxiety.
- Be proud of yourself for persevering and trying again.

Remember:

- True strength lies in the courage to keep going.
- Challenges are opportunities to sharpen your skills, strengthen your determination, and foster growth.
- For most people, success is rarely smooth: it comes through persistence, reflection, and continuous effort.

**Growing and persevering after failing an exam**

*Even accomplished professionals know what it's like to experience setbacks.* **Kukhu Prasetyo**, *an ophthalmologist, recalls a time when he faced a major exam failure – and what he learned from it.*

My initial objective for attempting the Ophthalmology Foundation examination was to assess my ophthalmological knowledge in a global context. Since international exams for ophthalmologists are still quite new in my country, I chose to lean only on my family and my mentor for support. From past experiences, I expected some people might doubt my goals, and I didn't want that to affect my peace of mind.

During my first attempt, my reading was quite cursory, and I didn't engage deeply with the material; however I was confident in my responses during the exam. When the results came out, and I had failed, I was (understandably) disheartened. To cope with the disappointment, I decided to be open about the outcome. I shared the results with my parents and my mentor. Their supportive words, reminding me that setbacks are part of the journey, brought me considerable comfort.

I believe that, in my situation, a prompt re-examination is the best strategy, as delaying might not improve my readiness, given my professional commitments. This time, I am doing my best to maintain a constructive perspective, viewing this as an opportunity to intensify my studies and enhance my professional capabilities. My main challenge before was a lack of focus and last- minute panic, so I am now working on self-discipline and better study habits. My strategy for this attempt also involves a comprehensive review of the recommended textbooks.

I wish all candidates the very best in in their examination. For those retaking the test, I admire your perseverance and encourage you to reflect on your original goals.

As for me, I will continue my studies while balancing my work and personal life. On the day of the test, I plan to pace myself in order to avoid past pitfalls. Afterwards, I intend to take a few days to rest and recharge – and perhaps even enjoy a brief visit to my hometown.

**Figure:**
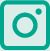
@ophthalmology.foundation

**Figure:**
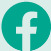
@Ophthalmology Foundation

**Figure:**
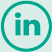
@Ophthalmology Foundation

**Figure:**
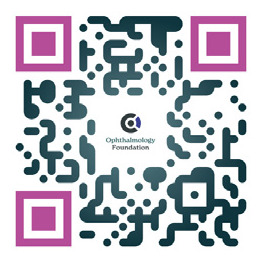


*The content of this page is supported by the Ophthalmology Foundation*

